# A phase I study of the HDM2 antagonist SAR405838 combined with the MEK inhibitor pimasertib in patients with advanced solid tumours

**DOI:** 10.1038/s41416-018-0355-8

**Published:** 2018-12-26

**Authors:** Vincent A. de Weger, Maja de Jonge, Marlies H. G. Langenberg, Jan H. M. Schellens, Martijn Lolkema, Andrea Varga, Brigitte Demers, Koruth Thomas, Karl Hsu, Gilles Tuffal, Samantha Goodstal, Sandrine Macé, Eric Deutsch

**Affiliations:** 1 Department of Clinical Pharmacology, The Netherlands Cancer Institute, Amsterdam, The Netherlands; 2 Department of Medical Oncology, Erasmus MC/Daniel den Hoed Cancer Center, Rotterdam, The Netherlands; 3 UMC Utrecht Cancer Center, Utrecht, The Netherlands; 4 Utrecht Institute for Pharmaceutical Sciences, Utrecht, The Netherlands; 5 Gustave Roussy, INSERM 1030, F-94805 Villejuif, France; 6 Sanofi Oncology, Vitry-sur-Seine, France; 7 Sanofi Oncology, Cambridge, USA; 8 Sanofi R&D, Montpellier, France; 9 EMD Serono Research & Development Institute, Inc., Billerica, USA; 10 University Paris-Sud, University Paris-Saclay, F-94270 Le Kremlin-Bicêtre, France

**Keywords:** Targeted therapies, Outcomes research

## Abstract

**Background:**

This phase I, open-label, dose-escalation study evaluated the safety, pharmacokinetics and pharmacodynamics of combination therapy with the HDM2 inhibitor SAR405838 and the MEK1/2 inhibitor pimasertib administered orally once daily (QD) or twice daily (BID) in locally advanced or metastatic solid tumours (NCT01985191).

**Methods:**

Patients with locally advanced or metastatic solid tumours with documented wild-type *TP53* and *RAS* or *RAF* mutations were enroled. A 3 + 3 dose-escalation design was employed. The primary objective was to assess maximum tolerated dose (MTD).

**Results:**

Twenty-six patients were treated with SAR405838 200 or 300 mg QD plus pimasertib 60 mg QD or 45 mg BID. The MTD was SAR405838 200 mg QD plus pimasertib 45 mg BID. The most common dose-limiting toxicity was thrombocytopenia. The most frequently occurring treatment-related adverse events were diarrhoea (81%), increased blood creatine phosphokinase (77%), nausea (62%) and vomiting (62%). No significant drug–drug interactions were observed. The biomarkers MIC-1 and pERK were, respectively, upregulated and downregulated in response to study treatment. In 24 efficacy-evaluable patients, one patient (4%) had a partial response and 63% had stable disease.

**Conclusions:**

The safety profile of SAR405838 and pimasertib combined was consistent with the safety profiles of both drugs. Preliminary antitumour activity was observed.

## Introduction

The tumour suppressor p53 has a pivotal role in preventing tumourigenesis through the induction of cell-cycle arrest and apoptosis.^1^ *TP53* is the most frequently mutated gene in human cancer; however, some tumours still harbour wild-type *TP53*.^2^ In these cases, wild-type *TP53* function is usually inhibited by the negative regulator mouse double minute 2 (MDM2, HDM2 in humans). HDM2 binds to the transactivation domain of p53 and acts as an E3 ubiquitin ligase, leading to degradation in the proteasome. Overexpression of HDM2 has been reported in various tumour types, and small-molecule inhibitors of HDM2 have demonstrated antitumour activity in preclinical studies.^3,4^

SAR405838 is an oral, selective spirooxindole derivative antagonist of HDM2.^4^ SAR405838 treatment results in p53 pathway activation, leading to p53-dependent cell-cycle arrest and apoptosis in preclinical models.^4^ SAR405838 monotherapy was investigated in a phase I dose-escalation study in patients with solid tumours, including a maximum tolerated dose (MTD) expansion cohort of patients with de-differentiated liposarcoma.^5^ The main dose-limiting toxicity (DLT) observed was thrombocytopenia; the MTD and recommended phase II dose of SAR405838 was 300 mg once daily (QD).^5^ In the phase I study, it was also shown that *TP53* mutations appeared in circulating cell-free DNA (cfDNA) from patients being treated over time with SAR405838. Furthermore, *TP53* mutation burden increased over time and correlated with change in tumour size, suggesting emergence of resistance to HDM2 inhibition.^6^

MEK is a key component of the mitogen-activated protein kinase (MAPK) signalling pathway, which is integral to the proliferation and survival of cancer cells.^7^ Activation of the GTPase RAS leads to a phosphorylation cascade via the kinases RAF, MEK and ERK that results in the activation of oncogenic gene expression. Activating mutations in the upstream components B-RAF or RAS (KRAS, NRAS and H-RAS) are the most frequent cause of upregulation of the MAPK signalling pathway.^8^ However, targeting RAS directly has been unsuccessful to date. Therefore, strategies for inhibiting the MAPK pathway have focused on inhibiting B-RAF and MEK (MEK1 and MEK2 isoforms). MEK inhibitors have demonstrated encouraging activity in preclinical studies and preliminary clinical activity in solid tumours exhibiting RAS pathway activation.^9^

Pimasertib is an oral, selective, small-molecule inhibitor of MEK1/2. In a phase I dose-escalation trial, DLTs included skin rash, acneiform dermatitis, ocular events and stomatitis.^10^ The MTD for pimasertib monotherapy using either a QD or a twice daily (BID) regimen is 90 mg and 60 mg, respectively; the recommended phase II dose of pimasertib monotherapy was determined at 60 mg BID. Pimasertib was investigated in phase I/II clinical trials in a number of tumour types, and has demonstrated preliminary clinical activity as monotherapy and in combination with other agents.^11,12^

Together, the p53 and MAPK pathways are the most frequently mutated tumour suppressor and oncogene pathways. Preclinical studies have provided rationale to test the combination of SAR405838 and pimasertib in tumours with wild-type *TP53* and MAPK pathway activation. In preclinical RAS pathway-activated, *TP53* wild-type xenograft melanoma models (UACC62), a therapeutic benefit was observed for the SAR405838 and pimasertib combination over the activity of either single-agent; durable tumour regression was observed with the combination.^13^

This phase I, dose-escalation study evaluated the safety, pharmacokinetics (PK) and pharmacodynamics (PD) of SAR405838 combined with pimasertib administered QD or BID in patients with locally advanced or metastatic solid tumours.

## Materials and methods

### Study design

This was a phase I, open-label, dose-escalation, safety, PK and PD study of SAR405838 administered QD and combined with pimasertib administered either QD or BID in 21-day cycles in adult patients with advanced solid tumours (NCT01985191). Both study medications were administered orally using a gelatine capsule formulation. Patients fasted for 2 h prior to and 1 h after each dose. Each dose of SAR405838, except at Cycle 1 Day 1 (C1D1), was to be taken immediately after pimasertib administration, preferably in the morning of each day. Treatment could have continued until precluded by toxicity, incompliance, progression or death.

The primary endpoint was MTD, as assessed by DLT, of SAR405838 and pimasertib combination therapy in patients with locally advanced or metastatic solid tumours. Secondary endpoints included safety, PK, PD and tumour response, as well as determination of the impact of study combination regimen on the genetic status of *TP53/RAS* when compared with baseline.

### Patient population

Patients eligible for inclusion were ≥ 18 years of age, with a histologically or cytologically confirmed solid tumour with documented wild-type *TP53* and *RAS/RAF* mutations for which no further effective standard treatment was available. Eligible patients had locally advanced or metastatic disease with at least one measurable lesion defined by Response Evaluation Criteria in Solid Tumours (RECIST) Version 1.1,^14^ an Eastern Cooperative Oncology Group performance status (ECOG PS) of 0–1, life expectancy ≥ 12 weeks and sufficient bone marrow function.

### Dose escalation and dose-limiting toxicities

A 3 + 3 design was used for dose escalation. The starting dose was SAR405838 200 mg QD/pimasertib 45 mg BID. Cohorts of three to six patients were enroled sequentially in ascending dose levels (DLs) per the protocol and decisions of the Study Committee (Investigators and Sponsor) based on the occurrence of DLTs within the first two cycles. Patients should have taken at least 80% of each study medication in order to be evaluable for DLT, unless precluded by the occurrence of a DLT. After confirmation of safety at the first DL1 of SAR405838 200 mg QD/pimasertib 45 mg BID, dose escalation was pursued independently and concomitantly according to the schedule of pimasertib. Using the pimasertib QD schedule, dose escalation was to sequentially proceed to DL2a (SAR405838 200 mg QD/pimasertib 60 mg QD) then DL3a (SAR405838 300 mg QD/pimasertib 60 mg QD). Using the pimasertib BID schedule, dose escalation was to sequentially proceed to DL2b (SAR405838 300 mg QD/pimasertib 45 mg BID) then DL3b (SAR405838 300 mg QD/pimasertib 60 mg BID). If one of three patients experienced a DLT in the first two cycles, the cohort was expanded to six patients for confirmation. If a DLT was observed in at least two out of a maximum of six patients at a DL, this was considered the maximum administered dose. The MTD was the highest DL where at most one patient of the cohort experienced a DLT.

A DLT was defined as any of the following drug-related adverse events (AEs) occurring during the first two cycles of treatment (Days 1–42): an AE that in the opinion of the safety committee was of potential clinical significance such that further dose escalation would expose patients to unacceptable risk; any grade ≥ 3 non-haematological toxicity (excluding Grade 3 fatigue persistent for < 7 days, Grade 3 vomiting or diarrhoea if controlled within 2 days with adequate therapy, asymptomatic Grade 3 creatinine phosphokinase (CPK) elevation, Grade 3 aspartate aminotransferase/alanine aminotransferase elevations < 7 days in duration, Grade 3/4 alkaline phosphatase (ALP) elevations in the context of bone metastasis, or Grade 3 hypertension that can be controlled within a week with oral antihypertensives); any Grade ≥ 3 thrombocytopenia; any Grade 4 neutropenia or febrile neutropenia; any Grade 4 anaemia; retinal vein occlusion; left-ventricular ejection fraction (LVEF) decrease > 20% from baseline or a decrease > 10% if baseline ejection fraction is 50%; Hy’s law; any treatment delay > 2 weeks owing to drug-related adverse effects; any severe or life-threatening complication or abnormality not defined in National Cancer Institute Common Terminology Criteria for Adverse Events (NCI-CTCAE) that is attributable to the therapy; and any toxicities resulting in an inability to complete at least 80% of planned trial medication doses during the first two cycles.

### Safety assessments

Safety was assessed by the evaluation of AEs, DLTs, changes in vital signs, ECOG PS, physical examinations,12-lead electrocardiograms, determination of LVEF, ophthalmological examinations and clinical laboratory tests (including haematology, coagulation, blood chemistry and urinalysis). AEs were graded according to NCI-CTCAE version 4.03.^15^

### Pharmacokinetic assessments

Blood samples were collected on Days 1 (for pimasertib only), 2, 3, 8 and 15 of Cycle 1, on Days 1 and 2 of Cycle 2, and on Day 1 of Cycles 3 and 4, to determine the whole-blood and plasma concentrations of SAR405838 and pimasertib, respectively. Calculation of PK parameters included maximum concentration (*C*_max_), time to reach maximum concentration (*t*_max_), time corresponding to the last concentration above the lowest limit of quantification (*t*_last_), area under the concentration-versus-time curve (AUC) from time 0 to time *t* (AUC_0–*t*_; 12 h for BID or 24 h for QD), AUC from time 0 to *t*_last_ (AUC_last_), AUC extrapolated to infinity, AUC over the dosing interval (AUC_tau_) and terminal half-life (*t*_1/2z_).

### Pharmacodynamic assessments

Blood samples for peripheral PD biomarkers analyses for both SAR405838 and pimasertib, including macrophage inhibitory cytokine-1 (MIC-1) levels in plasma and phosphorylated extracellular signal-regulated kinases (pERK) levels in peripheral blood mononuclear cells (PBMCs), were collected on Days 1, 2, 3, 8 and 15 of Cycle 1, on Days 1 and 2 of Cycle 2, and Day 1 of Cycles 3 and 4. MIC-1 protein concentrations were measured in plasma samples using an analytically validated enzyme-linked immunosorbent assay (Quantikine^®^ Human GDF-15 immunoassay). pERK levels were determined by evaluating median value changes in response to ex vivo stimulation with or without phorbol myristate acetate (PMA) over time in whole-blood samples (CD 45^+^ and lymphocyte populations) by flow cytometry (BD FACSCanto™ II instrument). Stimulated pERK levels were calculated as percentage (PMA-dimethylsulphoxide [DMSO])/DMSO).

### Molecular profiling assessments

#### Plasma preparation and cfDNA isolation from plasma

Plasma was prepared at clinical sites within 15–30 minutes from blood draw using double centrifugation as previously described.^6^ Blood samples were processed first by centrifugation at 1600 ( + 150) g for 10 minutes. The supernatant was transferred to a fresh 2 mL tube and was centrifuged again at 3000 ( + 150) g for 10 minutes. The resulting supernatant was transferred into a 3.5 mL polypropylene tube and stored at − 80 °C until cfDNA isolation. This process typically yielded ~ 1.2 mL of plasma for DNA isolation. For cfDNA isolation, we used the QIAamp^®^ Circulating Nucleic Acid Kit (QIAGEN, Catalogue # 55114) using the QIAvac 24 Plus (QIAGEN, Catalogue # 19413) according to the manufacturer’s recommended procedures.

#### Targeted sequencing library preparation and mutation analysis

In order to monitor tumour genetic status using liquid biopsies, we developed a targeted deep-sequencing assay for mutation detection based on a hybrid-capture target enrichment strategy.^6^ Mutation analysis was done as previously described.^6^

Assessment of tumour genetic status of *RAS/RAF* and *TP53* at baseline was performed at each site on either archival tissues (20 including 10 diagnostic specimen) or freshly biopsied tissues during screening (six patients).

### Efficacy assessments

Radiological tumour assessments were made at least every two cycles or less frequently, if indicated. Tumour response was investigator assessed using RECIST 1.1. A partial response (PR) or complete response must have been confirmed on a second examination performed at least 4 weeks apart in order to be documented as a confirmed response.

## Results

### Patient population

Twenty-six patients with locally advanced or metastatic tumours were treated (first patient enroled: 03 December 2013; last patient completed: 15 February 2016): DL1 SAR405838 200 mg QD/pimasertib 45 mg BID (*n* = 7); DL2a SAR405838 200 mg QD/pimasertib 60 mg QD (*n* = 4); DL2b SAR405838 300 mg QD/pimasertib 45 mg BID (*n* = 7); and DL3a SAR405838 300 mg QD/pimasertib 60 mg QD (*n* = 8).

Patient baseline characteristics are shown in Table 1. Median age was 59.5 years (range 45–79 years). Patients had advanced cancer diagnoses, most commonly colorectal (42%) or lung (31%). Median number of prior anticancer therapies was 3 (range 1–7). Twenty-one of the 26 (81%) patients discontinued the study owing to disease progression and five (19%) discontinued owing to AEs.

**Table 1:** Patient baseline characteristics

	All patients
	(*N* = 26)
Median age, years (range)	59.5 (45–79)
Male, *n* (%)	16 (62)
ECOG PS, *n* (%)	
0	10 (38)
1	16 (62)
Tumour type, *n* (%)	
Colorectal	11 (42)
Lung	8 (31)
Melanoma	1 (4)
Other	6 (23)
Median number of prior anticancer therapies (range)	3 (1–7)
Tumour molecular status, *n* (%)	
KRAS mutations	24 (92)
BRAF mutations	1 (4)
NRAS mutations	1 (4)

*ECOG PS* Eastern Cooperative Oncology Group performance status

### Dose escalation and dose-limiting toxicities

Three of 12 DLT-evaluable patients treated with the BID-pimasertib regimen experienced a DLT: one of six evaluable patients experienced two DLTs at DL1 (Grade 2 pustular rash and Grade 2 thrombocytopenia that led to dose interruptions and <  80% of dose completion) and two of six evaluable patients experienced one DLT at DL2b (one patient with Grade 2 thrombocytopenia, for which the SAR405838 dose was interrupted and not resumed, and one patient with Grade 4 increased lipase). DL3b was not tested.

One of eight DLT-evaluable patients treated with the QD pimasertib regimen experienced a DLT. No DLT occurred in three DLT-evaluable patients treated at DL2a, and one of five DLT-evaluable patients experienced a DLT at DL3a (Grade 3 thrombocytopenia). At that same DL, an AE meeting DLT definition (Grade 2 thrombocytopenia) occurred in Cycle 4, post-DLT evaluation period, which led to discontinuation of study medication in an additional patient. A sixth patient was not recruited to complete the cohort as DL3a was considered not tolerated.

The MTD was SAR405838 200 mg QD plus pimasertib 45 mg BID (highest total daily dose of pimasertib).

### Safety

Mean duration of treatment was 21 weeks (seven cycles per patient) for SAR405838 and pimasertib. The most frequently occurring AEs regardless of causality were diarrhoea (81%), increased blood CPK (77%), vomiting (73%), nausea (69%) and fatigue (58%; Supplementary Table 1). The most frequently occurring Grade ≥  3 AEs regardless of causality were pulmonary embolism, fatigue and thrombocytopenia (15% each; Supplementary Table 1). The most frequently occurring treatment-related AEs were diarrhoea (81%), increased blood CPK (77%), nausea (62%) and vomiting (62%; Table 2). The most frequently occurring Grade ≥ 3 treatment-related AEs were increased blood CPK (12%), fatigue (12%) and thrombocytopenia (12%; Table 2).

**Table 2:** Treatment-related AEs (related to SAR405838 and/or pimasertib) of any grade occurring in > 20% of patients and all treatment-related Grade ≥ 3 AEs

Preferred term, n (%)	DL1 SAR405838 200 mg QD + pimasertib 45 mg BID (n = 7)	DL2a SAR405838 200 mg QD + pimasertib 60 mg QD (n = 4)	DL2b SAR405838 300 mg QD + pimsertib 45 mg BID (n = 7)	DL3a SAR405838 300 mg QD + pimasertib 60 mg QD (n = 8)	All patients (N = 26)
Any treatment-related AE	7 (100)	4 (100)	7 (100)	8 (100)	26 (100)
Diarrhoea	6 (86)	2 (50)	7 (100)	6 (75)	21 (81)
Blood CPK increased	6 (86)	3 (75)	6 (86)	5 (63)	20 (77)
Nausea	2 (29)	4 (100)	4 (57)	6 (75)	16 (62)
Vomiting	5 (71)	3 (75)	4 (57)	4 (57)	16 (62)
Oedema peripheral	5 (71)	2 (50)	3 (43)	1 (13)	11 (42)
Fatigue	2 (29)	1 (25)	4 (57)	2 (25)	9 (35)
Dermatitis acneiform	4 (57)	0	4 (57)	1 (13)	9 (35)
Decreased appetite	3 (43)	2 (50)	3 (43)	1 (13)	9 (35)
Ejection fraction decreased	4 (57)	1 (25)	3 (43)	1 (13)	9 (35)
Rash	2 (29)	2 (50)	2 (29)	2 (25)	8 (31)
Retinal detachment	4 (57)	0	4 (57)	0	8 (31)
Rash pustular	2 (29)	1 (25)	2 (29)	2 (25)	7 (27)
Macular detachment	3 (43)	0	2 (29)	2 (25)	7 (27)
Stomatitis	4 (57)	1 (25)	0	2 (25)	7 (27)
Folliculitis	3 (43)	0	2 (29)	1 (13)	6 (23)
Any Grade ≥3 treatment-related AE	5 (71)	1 (25)	4 (57)	4 (50)	14 (54)
Blood CPK increased	2 (29)	0	1 (14)	0	3 (12)
Fatigue	1 (14)	0	1 (14)	1 (13)	3 (12)
Thrombocytopenia	0	0	1 (14)	2 (25)	3 (12)
Amylase increased	0	1 (14)	1 (25)	0	2 (8)
Lipase increased	1 (14)	0	1 (14)	0	2 (8)
Diarrhoea	0	0	0	1 (13)	1 (4)
Dermatitis acneiform	1 (14)	0	0	0	1 (4)
Ejection fraction decreased	1 (14)	0	0	0	1 (4)
Oral fungal infection	0	0	1 (14)	0	1 (4)
Pneumonitis	0	0	1 (14)	0	1 (4)
Stomatitis	1 (14)	0	0	0	1 (4)
Cheilitis	1 (14)	0	0	0	1 (4)
Aphthous ulcer	1 (14)	0	0	0	1 (4)
Palmar-plantar erythrodysesthesia syndrome	1 (14)	0	0	0	1 (4)
Asthenia	0	0	1 (14)	0	1 (4)

*AE* adverse event, *BID* twice daily, *CPK* creatinine phosphokinase, *QD* once daily

AEs of interest included increased blood CPK (77%; Grade  ≥  3 12%), decreased ejection fraction (38%; Grade  ≥ 3 4%), retinal detachment (31%; no Grade  ≥  3), macular detachment (27%; no Grade  ≥  3), thrombocytopenia (19%; Grade  ≥  3 15%) and increased troponin T (19%; no Grade  ≥  3). The most common Grade  ≥  3 haematological laboratory abnormality was lymphocyte count decreased (19%); the most common Grade  ≥  3 biochemistry laboratory abnormality was increased ALP (15%).

Fourteen patients (54%) had a serious AE (SAE), the most frequently occurring of which were disease progression (12%), constipation (8%) and accidental overdose (8%). Four patients (15%) had a treatment-related SAE, including macular detachment, pneumonitis, diarrhoea, nausea, vomiting and accidental overdose (4% each). Five patients (19%) had an AE leading to permanent treatment discontinuation, including rash pustular, dyspnoea, nausea, vomiting, increased blood CPK and ECG T-wave inversion (4% for each), and fatigue (8%). In addition, 17 patients had SAR405838 dose modification, reduction or omission, and 18 patients had pimasertib dose modification, reduction or omission. Four patients died during the study, with three deaths occurring in the post-treatment period. All deaths were owing to disease progression with no treatment-related deaths reported.

### Pharmacokinetics

**Fig. 1:**
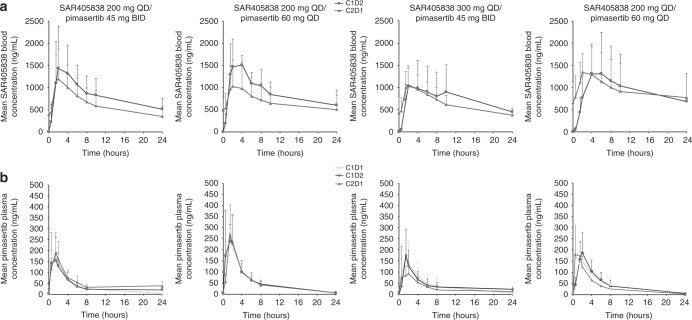
Mean plasma concentration–time profiles for **a** SAR405838 and **b** pimasertib. BID twice daily, C cycle, D day, QD once daily

Table 3 summarises SAR405838 and pimasertib PK parameters. Figure 1 shows SAR405838 and pimasertib concentration–time profiles. PK of SAR405838 and pimasertib when administered in combination was similar to the PK of SAR405838 or pimasertib when administered as monotherapy. Owing to respective drug variability, only a substantial drug–drug interaction may have been evidenced.

**Table 3:** Summary of PK parameters for SAR405838 and pimasertib when administered in combination

Cohort	Cycle/day	SAR405838 PK parameter, median (range) (*n*) or mean ± SD (*n*; CV%)							
		*t*_max_, *h*	C_max_, ng/mL	*C*_max_, Rac		AUC_0–24_, ng.h/mL	AUC_0–24_, Rac		*t*_1/2_, *h*
DL1	C1D2	2 [1.5–6] (7)	1570 ± 876 (7; 56)	0.71 ± 0.31 (6; 43)		19,300 ± 8570 (7; 44)	0.68 ± 0.27 (6,40)		16.1 ± 7.75 (3; 48)
	C2D1	1.8 [1.5–4] (6)	1250 ± 668 (6; 54)	–		14,600 ± 7080 (6; 49)	–		10.8 (2)
DL2a	C1D2	2 [1.5–6] (3)	1500 ± 634 (3; 42)	0.97 ± 0.4 (3; 42)		20,800 ± 7540 (3; 36)	1.1 ± 0.53 (3,50)		8.86 (1)
	C2D1	2.8 [1.5–4] (4)	1050 ± 581 (4; 55)	–		15,600 ± 8720 (4; 56)	–		10.0 (1)
DL2b	C1D2	3 [2–4] (6)	1100 ± 547 (6; 50)	0.92 ± 0.37 (5; 41)		16,300 ± 10,300 (6; 63)	1.1 ± 0.83 (5,77)		15.5 ± 4.33 (3; 28)
	C2D1	1.5 [0–2] (6)	1100 ± 361 (6; 33)	–		15,000 ± 4650 (6; 31)	–		13.4 ± 2.38 (3; 18)
DL3a	C1D2	4 [4–24] (7)	1490 ± 939 (7; 63)	1.3 ± 0.54 (5; 41)		21,400 ± 13,500 (7; 63)	1.3 ± 0.68 (5,53)		13.3 ± 4.94 (3; 37)
	C2D1	2 [1.5–4] (6)	1440 ± 583 (6; 40)	–		21,200 ± 13,700 (6; 64)	–		14.9 ± 11.1 (3; 74)
**Cohort**	**Cycle/day**	**Pimasertib PK parameter, median (range) (** ***n*** **) or mean ± SD (** ***n*** **; CV%)**							
		***t***_max_, ***h***	***C***_**max**_ **, ng/mL**	***C***_max_, Rac^a^	***C***_max_, combination effect^a^	**AUC**_**0–12**_ **, ng.h/mL**	**AUC**_**0–12**_ **, Rac** ^**a**^	**AUC**_**0–12**_ **, combination effect** ^**a**^	***t***_1/2_, ***h***
DL1	C1D1	0.5 [0.5–2] (7)	218 ± 108 (7; 49)	1.1 ± 0.28 (6; 25)	1.1 ± 0.78 (7; 73)	755 ± 382 (7; 51)	1.2 ± 0.3 (6; 26)	0.94 ± 0.21 (7; 22)	4.76 ± 1.17 (7; 24)
	C1D2	1.5 [0.5–2] (7)	200 ± 102 (7; 51)	–	–	689 ± 324 (7; 47)	–	–	NR
	C2D1	1.5 [0.5–1.5] (6)	226 ± 70.6 (6; 31)	–	–	862 ± 350 (6; 41)	–	–	NR
DL2a	C1D1	1.5 [0.5–1.5] (4)	301 ± 69.9 (4; 23)	0.97 ± 0.39 (4; 41)	0.97 ± 0.24 (4; 24)	1120 ± 410 (4; 37)	0.95 ± 0.34 (4; 36)	1 ± 0.13 (4;12)	5.38 ± 0.276 (4; 5)
	C1D2	1 [0.5–2] (4)	303 ± 134 (4; 44)	–	–	1130 ± 422 (4; 37)	–	–	NR
	C2D1	1.5 [1.5–-2] (4)	269 ± 134 (4; 50)	–	–	1040 ± 504 (4; 48)	–	–	NR
DL2b	C1D1	1.5 [0.5–1.5] (7)	184 ± 109 (7; 59)	1.2 ± 1.1 (6; 87)	0.99 ± 0.25 (7; 26)	779 ± 594 (7; 76)	0.94 ± 0.36 (6; 38)	0.95 ± 0.17 (7; 18)	4.71 ± 1.39 (7; 30)
	C1D2	1.5 [1.5–2] (7)	182 ± 110 (7; 61)	–	–	684 ± 367 (7; 54)	–	–	NR
	C2D1	1.5 [0.5–2] (6)	147 ± 93.8 (6; 64)	–	–	532 ± 166 (6; 31)	–	–	NR
DL3a	C1D1	1 [0.5–4] (8)	221 ± 98.3 (8; 44)	1.8 ± 1.1 (4; 61)^b^	1.1 ± 0.41 (7; 39)	935 ± 469 (8; 50)	1.1 ± 0.25 (4; 24)	1.1 ± 0.16 (7; 14)	5.25 ± 1.83 (7; 35)
	C1D2	1.5 [0.5-2] (7)	200 ± 84.4 (7; 42)	–	–	917 ± 390 (7; 43)	–	–	NR
	C2D1	1.5 [0.5–1.5] (5)	243 ± 65.4 (5; 27)	–	–	765 ± 314 (5; 41)	–	–	NR

^a^Rac = C2D1 vs C1D2 ratio; combination effect = C1D2 vs C1D1 ratio ^b^Mean Rac high owing to two patients exhibiting low *C*_max_ on C1D1

*AUC* area under the concentration-versus-time curve, *C* cycle, C_*max*_ maximum concentration, *CV* coefficient of variation, *D* day, *DL* dose level, *NR* not reported, *PK* pharmacokinetic, *SD* stable disease, t_*1/2*_ terminal, t_*max*_ time to reach maximum concentration

The dose increase (50%) between DL1/DL2a (SAR405838 200 mg) and DL2b/DL3a (SAR405838 300 mg) did not result in SAR405838 exposure increase. The dose increase (33%) between DL1/DL2b (pimasertib 45 mg BID) and DL2a/DL3a (pimasertib 60 mg BID) resulted in pimasertib exposure increase.

### Pharmacodynamics

**Fig. 2:**
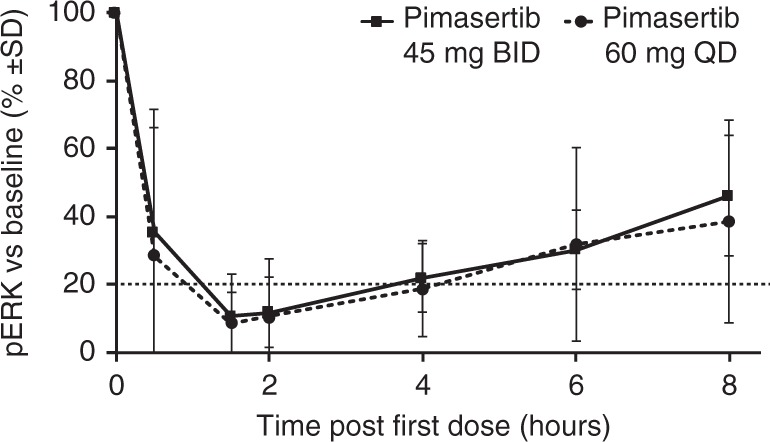
Inhibition of pERK in PMA‑stimulated PBMCs at C1D1 (*n* = 26). BID twice daily, C cycle, D day, PBMC peripheral blood mononuclear cells, pERK phosphorylated extracellular signal-regulated kinases, PMA phorbol myristate acetate, QD once daily, SD stable disease

Inhibition of pERK was evaluated in PMA-stimulated PBMCs; ≥  80% pERK inhibition was observed at C1D1, Cycle 1 Day 2 (C1D2) and Cycle 2 Day 1 (C2D1), at or around the *t*_max_ of pimasertib, at most DLs, and was maintained for 4  h (Fig. 2). Pimasertib dosing at 45 mg and 60 mg induced similar inhibition, which was not affected by SAR405838 administration.

Induction of MIC-1, a non-tumour-specific soluble protein regulated by p53, was evaluated. MIC-1 elevation (mean 3.5+ /− 0.8-fold vs baseline) was observed in all patients at C1D2 and C2D1, peaking at 6 h on both days. SAR405838 dosing at 200 or 300 mg induced a similar increase, which was not affected by pimasertib administration (Supplementary Fig. 1). A patient with confirmed PR (endometrial tumour) had the greatest increase in MIC-1 (8.8‑fold) in the DL1 cohort.

### Molecular profiling

The mutation status of *TP53*, *RAS* family genes and *BRAF* in cfDNA derived from plasma of 25 patients (one sample missing) was used to correlate with that from tumour tissue at screening. There was a partial concordance between tumour tissue (data provided by the clinical sites) and plasma cfDNA data collected at screening. Sixteen of 25 plasma samples had detectable level of *KRAS* (*n* = 14), *BRAF* (*n* = 1) or *NRAS* (*n* = 1) mutations, which were 100% concordant to reported tumour DNA genotype. Three of 25 plasma samples had detectable mutations in *TP53*, with a frequency ranging from 0.35% to 18%.

Mutation status was also analysed to look at the emergence of mutations after study treatment. Samples from 13 patients were collected. De novo *TP53* mutations were only seen in two patients after study treatment, and this occurred after Cycle 3. Of the two patients with de novo *TP53* mutations, one had best response of SD and progressive disease at Cycle 6, the other had a PR at Cycle 2 and progressive disease at Cycle 12.

### Efficacy

**Fig. 3:**
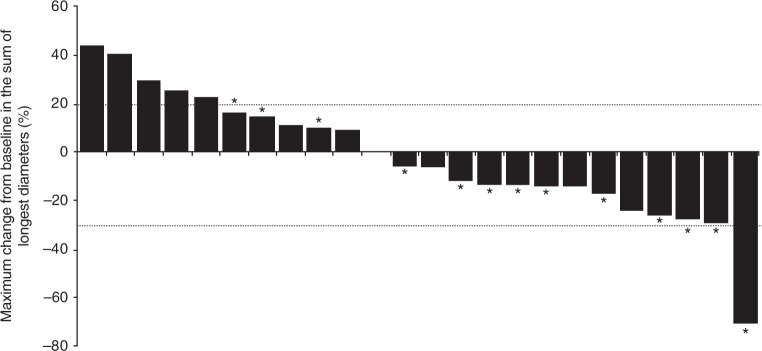
Maximum percentage change from baseline in the sum of target lesion diameters. Dotted lines represent threshold required for partial response (− 30%) and progressive disease (+ 20%). * Pimasertib BID. BID twice daily

In 24 efficacy-evaluable patients, the best overall response was PR for one patient (4%) with endometrial adenocarcinoma at SAR405838 200 mg QD/pimasertib 45 mg BID. Stable disease (SD) was the best overall response observed for 15 patients (63%), including patients with colorectal (seven patients), lung (five patients), pancreatic (one patient), intrahepatic bile duct (one patient) cancer and skin melanoma (one patient). Four patients had prolonged SD of  > 6 months (three patients with colorectal cancer and one patient with intrahepatic bile duct cancer). Eight patients (33%) had progressive disease as best response. Best percentage change in target lesion diameters is shown in Fig. 3; changes in target lesion diameters were variable and were generally not dose dependent; however, tumour shrinkage was mostly observed in the pimasertib BID-based regimens.

## Discussion

This phase I, dose-escalation study evaluated the safety, PK and PD of combination therapy with SAR405838 and pimasertib (QD and BID) in advanced solid tumours. The MTD was SAR405838 200 mg QD plus pimasertib 45 mg BID; the main DLT was thrombocytopenia. The safety observations of thrombocytopenia are consistent with other drugs in the HDM2 antagonist class,^16–20^ and the mechanism of action.^21^

At the MTD, significant dose interruptions and reductions occurred after Cycle 2 owing to late toxicities and poor tolerance. Single-agent MTDs of SAR405838 and pimasertib could not be administered in combination, owing to overlapping toxicity. The most common treatment-related AEs were diarrhoea and blood CPK increase. Compared with pimasertib BID regimen, the QD regimen induced less diarrhoea, vomiting, electrolytes imbalance, skin reaction, ocular events, CPK increase and drop in LVEF. In addition, AEs meeting DLT definition occurring late, and responsible for dose delay and reduction, occurred in patients receiving the BID-pimasertib schedule.

No significant drug–drug interactions were observed. Considering respective variability of SAR405838 and pimasertib, the PK profiles were generally consistent with previous data for each drug alone. Only a substantial drug–drug interaction may have been evidenced. PD effect of both agents was demonstrated at all doses tested. The PD biomarkers MIC-1 and pERK were, respectively, upregulated and downregulated in response to study treatment.

In a phase I monotherapy study in solid tumours, emergence of *TP53* mutations in patients being treated with SAR405838 was shown.^6^ In this combination study of SAR405838 +  pimasertib, emergence of *TP53* mutations during treatment was only seen in 2 of 13 patients tested, suggesting that combination with pimasertib may affect the emergence of *TP53* mutations and thus resistance to HDM2 antagonist. There was only 64% concordance for *KRAS* mutation presence between tumour tissue and plasma samples where *KRAS* mutations were reported in the tumour but not in the plasma samples. There were also some discrepancies for *TP53* mutations where three mutations were detected in plasma and not in the three patients’ tumour samples. This could be explained by the difference in date of collection for both samples or difference in assay sensitivity. For *KRAS* mutation discrepancies, tissue heterogeneity where a rare mutated clone would have been detected in a specific region of the tumour but not found in the plasma sample could also be a relevant explanation.

The best overall response was PR for one patient with an endometrial tumour. For the majority of patients (63%) the best overall response was SD. Prolonged SD ( > 6 months) was observed in three patients with colorectal cancer and one patient with intrahepatic bile duct cancer. The data suggest that pimasertib BID may have increased MAPK pathway inhibition and tumour shrinkage compared with the QD regimen.

In summary, this phase I dose-escalation study evaluated combination therapy with SAR405838 and pimasertib in locally advanced or metastatic solid tumours. SAR405838 and pimasertib could not be administered at the single-agent MTDs when combined. However, preliminary antitumour activity was observed, suggesting potential benefit of restoring p53 activity while inhibiting the MAPK pathway in *TP53* wild-type and MAPK-mutated malignancies.

## Electronic supplementary material

Supplemental material

## Data Availability

Data are available on request. Biomarker data were posted to EU registry in February 2017; all remaining samples were destroyed in January 2017. Raw PK data were archived electronically; all remaining samples were destroyed.
