# Supplementary material for: A phase I study of the HDM2 antagonist SAR405838 combined with the MEK inhibitor pimasertib in patients with advanced solid tumours
Source: Br J Cancer. 2018 Dec 26;120(3):286–93. doi: 10.1038/s41416-018-0355-8 (PMC6354023; doi:10.1038/s41416-018-0355-8)
Supplement: Supplementary file 1 — Supplemental material [file 41416_2018_355_MOESM1_ESM.docx]

**Supplementary Tables and Figures**

**Supplementary Table 1.** AEs of any grade, regardless of causality, occurring in >20% of patients and Grade ≥3 AEs occurring in >5% of patients

| **Preferred term, n (%)** | **All patients**  **N = 26** |
| --- | --- |
| **Any AE** | 26 (100) |
| Diarrhoea | 21 (81) |
| Blood CPK increased | 20 (77) |
| Vomiting | 19 (73) |
| Nausea | 18 (69) |
| Fatigue | 15 (58) |
| Decreased appetite | 13 (50) |
| Oedema peripheral | 12 (46) |
| Ejection fraction decreased | 10 (38) |
| Dermatitis acneiform | 9 (35) |
| Dry mouth | 9 (35) |
| Dyspnoea | 9 (35) |
| Rash | 8 (31) |
| Retinal detachment | 8 (31) |
| Asthenia | 7 (27) |
| Rash pustular | 7 (27) |
| Macular detachment | 7 (27) |
| Stomatitis | 7 (27) |
| Abdominal pain | 6 (23) |
| Dyspepsia | 6 (23) |
| Folliculitis | 6 (23) |
| Hypokalaemia | 6 (23) |
| **Any grade ≥3 AE** | 20 (77) |
| Pulmonary embolism | 4 (15) |
| Fatigue | 4 (15) |
| Thrombocytopenia | 4 (15) |
| Amylase increased | 3 (12) |
| Blood CPK increased | 3 (12) |
| Lipase increased | 3 (12) |
| Asthenia | 2 (8) |
| Disease progression | 2 (8) |
| Dyspnoea | 2 (8) |

*AE* adverse event, *CPK* creatine phosphokinase

**Supplementary Fig. 1** Induction of MIC-1 at steady state (between 1 and 3 weeks of administration) at 6 hours post dose in patients receiving pimasertib plus A) 200 mg and B) 300 mg SAR405838. Each point represents an individual patient


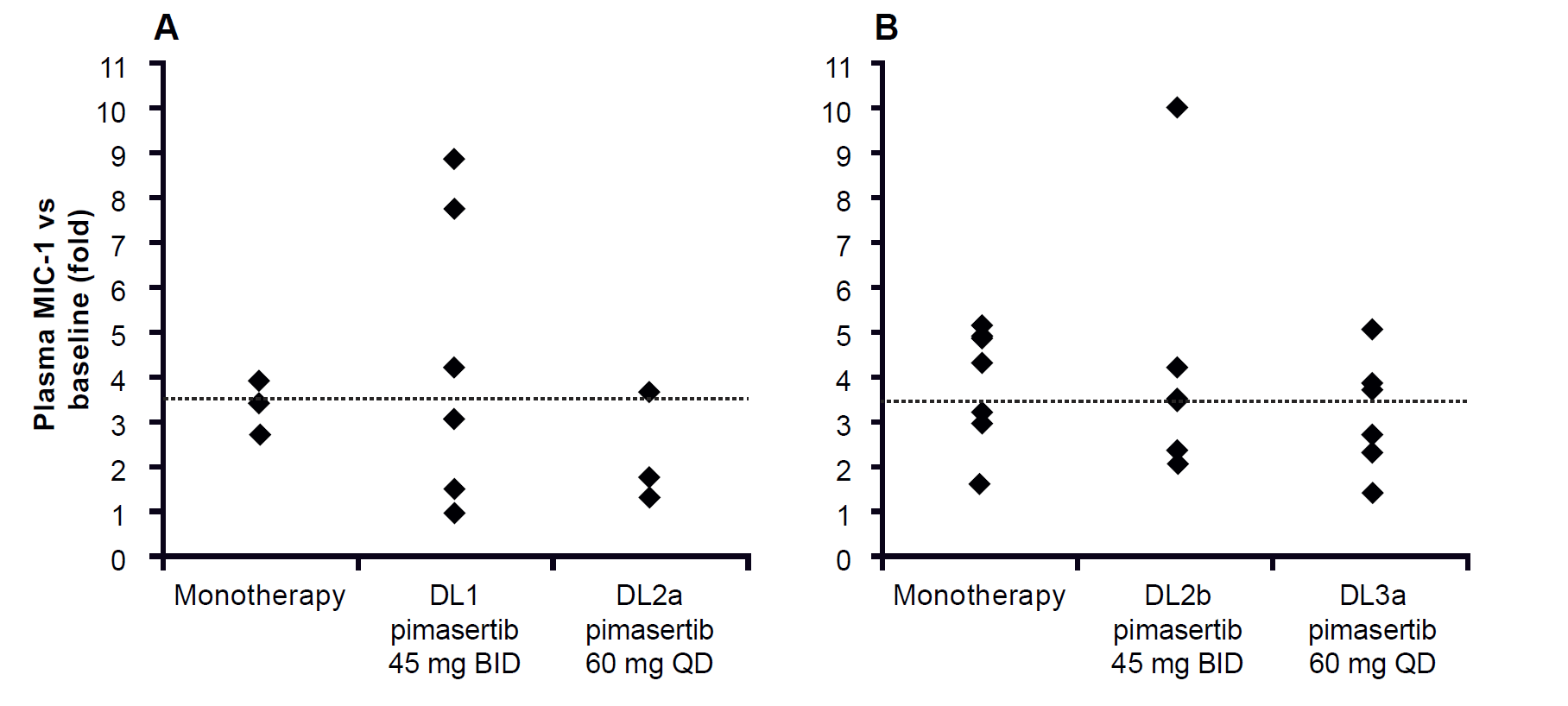


*BID* twice daily, *DL* dose level, *MIC-1* macrophage inhibitory cytokine 1, *QD* once daily
